# SAR Modeling to Predict Ames Mutagenicity Across Different *Salmonella typhimurium* Strains

**DOI:** 10.3390/ph18121853

**Published:** 2025-12-04

**Authors:** Alexander V. Dmitriev, Alexey A. Lagunin, Anastasia V. Rudik, Polina I. Savosina, Dmitry S. Druzhilovskiy, Dmitry A. Filimonov, Vladimir V. Poroikov

**Affiliations:** 1 Department for Bioinformatics, Institute of Biomedical Chemistry, 119121 Moscow, Russia; alexey.lagunin@ibmc.msk.ru (A.A.L.); vladimir.poroikov@ibmc.msk.ru (V.V.P.); 2 Department for Bioinformatics, Pirogov Russian National Research Medical University, 117513 Moscow, Russia

**Keywords:** SAR modeling, Ames test mutagenicity prediction, PASS, structure-activity relationships, drug design, *Salmonella typhimurium*, computational toxicology

## Abstract

**Background:** The Ames test, a biological assay employing various strains of *Salmonella typhimurium*, serves as a cornerstone in genetic toxicology for evaluating the mutagenic and potentially carcinogenic properties of chemical compounds. However, experimental testing is resource-intensive and time-consuming for screening the vast chemical space of existing and novel drug candidates in pharmaceutical development. **Methods:** To address this limitation, we have developed the Ames Mutagenicity Predictor web application, which predicts mutagenic activity in the Ames test for given structural formulas across a comprehensive panel of different bacterial strains. The application utilizes advanced structure–activity relationship (SAR) models generated by PASS (Prediction of Activity Spectra for Substances) v2024 software. The training set comprised 3250 compounds with experimentally determined mutagenicity across 69 different strains, compiled from peer-reviewed literature and established databases, and 4285 non-mutagenic compounds from the WWAD as negative examples. **Results:** Leave-one-out cross-validation (LOOCV) of the 69 strain-specific models yielded an average Invariant Accuracy of Prediction (IAP) of about 0.944, and for the unspecified mutagenicity, a value of 0.962 was obtained. **Conclusions:** These validated models have been integrated into a freely accessible web application Ames Mutagenicity Predictor that enables users to input compound structures through multiple formats: a built-in chemical editor, SMILES notation, or compound name search. The application generates comprehensive reports detailing the predicted probability of positive Ames test results for each individual strain, providing researchers with detailed mutagenicity profiles.

## 1. Introduction

The Ames test is a bacterial reverse-mutation assay widely used to screen chemicals for genotoxic and carcinogenic potential [1]. The Ames test utilizes histidine-dependent strains of *Salmonella typhimurium* to identify chemical mutagens—compounds that induce reverse mutations—thereby restoring the bacteria’s capacity to synthesize histidine. In practice, a battery of strains is used, with each carrying specific genetic lesions, so that different classes of mutagens (base-pair versus frameshift agents) and metabolic effects are covered [2]. For example, standard protocols typically include the *Salmonella typhimurium* strains TA98, TA100, TA1535, TA1537 or TA97, TA102, etc. These strains carry mutations in different positions of the histidine operon and often involve additional lesions that increase cell wall permeability or reduce DNA repair capacity, thereby enhancing sensitivity [2]. Since certain compounds require mammalian metabolic activation to become mutagenic (pro-mutagens), the assay is normally run with and without rat-liver S9 metabolic fraction. A positive Ames result is considered a strong indicator of potential carcinogenicity, and indeed many known carcinogens give positive Ames tests. The test is widely used in regulatory toxicology (e.g., OECD TG 471) as a first-tier screen for genotoxic impurities, environmental chemicals, and drug candidates in pharmaceutical development. Notably, regulatory guidelines such as ICH M7 [3] explicitly encourage the use of advanced (Q)SAR and computational toxicology approaches to assess mutagenic potential, either to complement or, in some cases, replace initial in vitro testing.

Despite its utility, the Ames assay shows significant experimental variability: reported intra-laboratory and inter-laboratory reproducibility is on the order of 80–85% [4]. This reflects factors like biological noise, borderline responses, and differences in interpretation. Because obtaining experimental Ames data for new compounds is time-consuming and often inconsistent, in silico models have been developed to predict Ames mutagenicity based on chemical structures (often based on (quantitative) structure–activity relationship analysis (Q)SAR). These in silico predictions are increasingly accepted in regulatory frameworks as supporting evidence. However, most (Q)SAR studies have focused on a handful of standard strains or on consensus results, and the predictive performance typically plateaus around the level of experimental reproducibility [5,6].

Recent advances in computational mutagenicity prediction have demonstrated the necessity for developing strain-specific models, rather than relying on consensus or binary classifications. Previous (Q)SAR studies typically collapsed the heterogeneous responses across the Ames test battery into a single aggregated “mutagenic” or “non-mutagenic” outcome [6]. However, this approach obscures mechanistically important information. Moreover, (Q)SAR models found in the literature are generally trained using only overall values, i.e., toxic and nontoxic classes resulting from the Ames test, without considering the intermediate results achieved by the experiments individually conducted for each strain [7]. This reductionist approach has limited the predictive utility of existing models. For instance, Xu et al. [6] achieved external validation accuracies between 90.4 and 98.0% for binary classification, yet this high accuracy masks the inability to discriminate strain-specific responses. The multitask deep learning approach of Martínez et al. [7] represents a significant breakthrough, showing that MTL-DNN models yield substantially higher specificity compared with single-task overall-label models, precisely because they leverage the complementary information from five individual strains. Building on these insights, our approach extends this paradigm by developing strain-specific SAR models for 69 different strains—nearly tenfold more than previous multitask approaches—thereby providing comprehensive strain-level predictions that enable researchers to understand not only whether a compound is mutagenic, but precisely which bacterial variants are affected.

In this study, we aimed to address this gap by developing SAR models for a large number of strains (69) across 7535 compounds, aiming for strain-specific predictions of Ames test mutagenicity. We present the Ames Mutagenicity Predictor https://www.way2drug.com/ames-mutagenicity/ (accessed on 1 December 2025), a web application based on the PASS (Prediction of Activity Spectra for Substances) algorithm [8] that offers comprehensive and mechanistically informed predictions. Previously, a web application for rodent organ-specific carcinogenicity prediction was created based on PASS technology [9]. In this study, the high predictive accuracy of the individual strain models (average value of Invariant Accuracy of Prediction (IAP) is 0.944) ensures reliable results. This application is intended for use by medicinal chemists, toxicologists, and regulatory specialists to identify mutagenic hazards early in the development process, thereby reducing the reliance on animal testing and accelerating the design of safer chemicals.

## 2. Results

The final training set comprised 7535 unique chemical compounds. Following the training process, PASS generated 69 individual SAR models, each corresponding to a specific *Salmonella typhimurium* strain, and an unspecified (a compound was considered mutagenic if at least one strain showed this effect) mutagenicity model. As shown in Table 1, the predictive performance of these models is exceptionally high. For all 69 models, the average sensitivity is 0.896, the average specificity is 0.920, and the average IAP calculated using leave-one-out cross-validation (LOOCV) procedure is 0.944. For undetermined mutagenicity (3250 compounds), sensitivity and specificity values are 0.907, and IAP is 0.962. The IAP values are close to the results of five-fold cross-validation (5FCV) (Table 1). These high levels of accuracy demonstrate the robustness of the models and their suitability for reliable in silico screening. For 23 of the 69 strains, the sensitivity is 1, and the IAP is higher than 0.97, and for 22 of them the specificity is higher than 0.9. The canonical strain TA100 (NP = 2181, where NP is the number of positive examples) has a model with sensitivity, specificity, and IAP of 0.851, 0.850, and 0.912, whereas the TA102 model (NP = 206) has values of 0.786, 0.801, and 0.875, respectively. Other classical strains have models with similar accuracy: for example, TA1535 (NP = 467) has values of 0.827, 0.830, and 0.902, TA1537 (NP = 394) has values of 0.850, 0.851, and 0.913, and TA1538 (NP = 311) has values of 0.839, 0.836, and 0.911. In general, all models (strain-specific SARs) achieved sensitivity above 0.6 and IAP above 0.8, demonstrating strong structure–mutagenicity relationships in this dataset.

The validated SAR models were integrated into a user-friendly Ames Mutagenicity Predictor web application (https://way2drug.com/ames-mutagenicity/, (accessed on 1 December 2025). The application interface allows users to input a chemical structure by drawing it in the Marvin JS editor, entering a SMILES string, or conducting a search based on a common compound name. The prediction results are displayed in a sortable table, listing all 69 strains and unspecified mutagenicity (based on Pa > Pi cut-off), and the corresponding Pa and Pi values. Results can be filtered and downloaded in multiple formats (CSV, PDF, XLSX). The application is implemented in PHP 8.2.6 and jQuery 3.6.0, with the PASS 2024 prediction engine running on a dedicated server.

The user can select a threshold for the Pa value to define positive predictions. The results are presented in a table, allowing users to quickly identify which strains for a compound are predicted to be mutagenic. The results table is fully interactive; users can sort predictions by any column (Pa, Pi, or strain name). Additionally, an integrated search function enables rapid filtering of the results by strain name or activity type, facilitating identification of strain-specific responses for particular compounds of interest. To support informed interpretation of results, the web application includes a “Training Set” section that provides detailed information about the composition of the training dataset and statistical metrics of model performance. The “Interpretation” section offers practical guidelines for understanding Pa and Pi values, and appropriate thresholds for decision-making.

**Figure 1:**
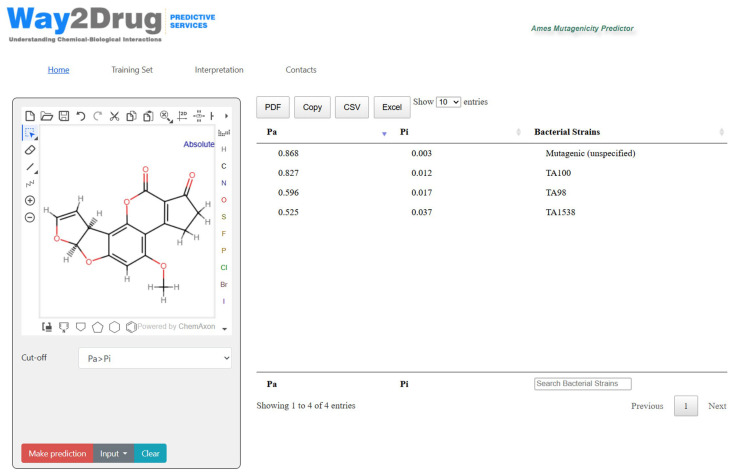
The interface of Ames Mutagenicity Predictor web application with the prediction results for Aflatoxin B1. The results table is ordered by the probability to be active (Pa). The prediction results are shown with cut-off Pa > Pi.

Figure 1 demonstrates the application’s interface and the prediction results for Aflatoxin B1, which is a known mutagen in *Salmonella typhimurium* TA100, TA98, TA1538 strains [10].

The results represented in Figure 1 demonstrate the following: the Ames Mutagenicity Predictor accurately identified aflatoxin B1 (AFB1) as a strain-specific mutagen, demonstrating strong concordance of predictions (using the cut-off Pa > Pi) with experimentally validated data from the Wheeler et al. study [10]. The models correctly predicted TA100 as the most responsive strain (Pa = 0.827, Pi = 0.012), followed by TA98 (Pa = 0.596, Pi = 0.017), and TA1538 (Pa = 0.525, Pi = 0.037). Notably, the model did not predict a mutagenic response for TA1535 (for this strain Pa < Pi), which experimental data [10] confirmed despite its structural similarity to the highly responsive TA100 strain. This strain-specific mutagenicity prediction exemplifies the model’s capability to distinguish between mutagenic and non-mutagenic responses. The prediction results also include “Mutagenic (unspecified)” activity with even higher Pa probability values compared to strain-specific mutagenicity. This may also serve as an additional criterion in favor of classifying this compound as a mutagen.

## 3. Discussion

Comprehensive validation metrics are provided in Table 1, where all possible accuracy estimates are reported, including both LOOCV and 5FCV results with confidence intervals. The consistency between the IAP obtained using the LOOCV and 5FCV procedures for all strains (see Table 1) confirms the reliability of the models; no significant drop in accuracy was observed under a more stringent validation scheme. For example, the TA100 model showed an IAP of 0.912 (LOOCV) vs. 0.910 with range 0.908–0.911 (5FCV), and the mutagenic (unspecified) model showed 0.962 vs. 0.960 with range 0.960–0.961, respectively. These minor differences indicate that the models are not overfitted and would generalize well to new compounds. In practice, the user can enter the chemical structure and obtain predicted results (probabilistic values of Pa and Pi) for each strain with confidence that ~95% of the predictions will correctly reflect the known mutagenic activity.

The large number and diversity of strains incorporated here is an important strength. By covering 69 strain–genotype variants, the models can capture compounds that are mutagenic via very specific mechanisms. It is well established that some chemicals induce frameshift mutations only in one strain and not in others, which underscores the critical importance of using standardized strain panels as mandated by international regulatory guidelines [3]. Our comprehensive, multi-strain SAR approach accommodates such specificities and exemplifies advanced chemoinformatics methodology in computational drug safety assessment. High-accuracy models for particular strains (e.g., GW257, TA1536) suggest that these screens were able to identify unique mutagenic patterns, even if based on few examples. Conversely, the TA100 model (the largest dataset) had relatively lower accuracy, possibly reflecting chemical diversity and experimental noise in its many examples. While the training set encompassed compounds tested both with and without S9 metabolic activation, the models operate without explicit S9-status discrimination, suggesting that incorporation of metabolic context as an additional parameter could further refine mechanistic predictions and will be a key area for improvement in future versions.

From a regulatory perspective, the performance of these SAR models is encouraging. In fact, Honma et al. estimated that the inherent ceiling for (Q)SAR accuracy is around 80–85% because of experimental variability [5]. The higher accuracy rates in our models, however, indicate that the in silico predictions are generally consistent with the majority of experimental data. According to international guidelines (OECD QSAR principles, ICH M7), such validated SAR models can be used as a first assessment of mutagenicity [3]. The availability of strain-specific models is particularly valuable: it allows a chemical to be profiled against each Ames variant, analogous to actually performing the multiflask assay in silico.

The models cover strains with high specificity. For instance, models for strains like TA100 (NP = 2181) and TA98 (NP = 1727), which are the most prevalent in the dataset and regulatory testing, show high accuracy and robustness (sensitivity, specificity > 0.85, IAP (LOOCV and 5FCV) > 0.91). Models for more specialized strains, often with fewer training examples, show the highest accuracy (for 23 strains with sensitivity 1 and specificity higher than 0.88; maximum NP value is 26 for TA1977), which can be explained by the high specificity of the compounds active for these strains and the performance of the PASS algorithm to handle such cases. In spite of high levels of accuracy, there may be some risks when using these models. Also, the underlying experimental data likely contains noise and some “false positives”/”false negatives”, which can limit even perfect algorithms. The PASS models inherently include a cut-off (Pa > Pi by default) to categorize activity. Borderline chemicals near this threshold may be incorrectly classified due to noise in the experimental data. Therefore, users should exercise caution when interpreting predictions for compounds with Pa values close to the discriminative threshold. Nonetheless, as a complementary tool, these SAR models can prioritize compounds for Ames testing or serve as weight-of-evidence in safety evaluations. The results of prediction given by the Ames Mutagenicity Predictor web application can complement and detail the results of other widely used web applications for assessing general Ames mutagenicity, such as pkCSM [11], Deep-PK [12], preADMET [13], Pro-Tox-II [14], and ADMETlab 2.0 [15], which lack mechanistic discrimination of the genotype-specific mutagenic potential conferred by distinct genetic backgrounds and repair capabilities among *Salmonella typhimurium* strains.

By providing predictions for each strain, the method can highlight specific mutagenic risks (e.g., if a compound is predicted positive only in TA102, which detects oxidative mutagens [16]) and guide further testing. Following the PASS methodology, we recommend prioritizing experimental validation according to the predicted Pa values in descending order; compounds with the highest Pa values should be tested first, proceeding step by step until the desired safety assessment goal is achieved. This strategy optimizes resource allocation and focuses experimental effort on the most likely hazards.

## 4. Materials and Methods

Dataset. The training set was created based on the data from peer-reviewed literature and established databases containing information on Ames test results. We extracted 3250 unique compounds that had been assayed for mutagenicity on various *Salmonella typhimurium* strains under standard conditions. No special selective filtering or curation was applied to our dataset. Therefore, its structural diversity reflects the variety of compounds examined in the original experimental studies. In total, 102 distinct strain records (including different genetic variants and metabolic activation states) were present. Among the strains, there were standard strains recommended by the OECD (e.g., TA100, TA98, TA1535, TA1537, TA102) as well as numerous engineered derivatives (e.g., YG1012, YG1041, TA7001-TA7006), which offer enhanced sensitivity towards specific mutagen classes. The strain names were considered as a class of activity of mutagenic compounds causing mutagenic effects for appropriate *Salmonella typhimurium* strains. Each data point represented the result (active/mutagenic or inactive/non-mutagenic) of a compound tested on a specific strain, taking into account the testing conditions (e.g., with or without metabolic activation S9) noted and integrated into the strain-specific activity definition were significant. Special class of activity, “mutagenic,” includes all compounds that are mutagenic to at least one strain. The structural formulas of the compounds were standardized and curated. The 4285 non-mutagenic compounds from the World Wide Approved Drugs database (WWAD) [17] were used as negative examples.

SAR Modeling with PASS. Models of the structure-activity relationship (SAR) for predicting the mutagenic activity for each of the 69 strains and unspecified mutagenicity were built using the PASS (Prediction of Activity Spectra for Substances) software [8]. PASS uses the Multilevel Neighborhoods of Atoms (MNA) descriptors and a Bayesian-like algorithm to predict multiple types of biological activity simultaneously. For this work, each “activity” was defined as the mutagenicity of a compound for a specific *Salmonella typhimurium* strain (e.g., “TA100 Mutagen”, “TA98 Mutagen”), and special class of activity, “mutagenic,” includes all compounds that are mutagenic to at least one strain. So, PASS prediction results with a list of *Salmonella typhimurium* strains may be considered as a profile of bacterial mutagenicity.

The training procedure included leave-one-out cross-validation (LOOCV) and 5-fold cross-validation (5FCV) procedures to estimate the prediction accuracy for each activity type. The 5FCV procedures were repeated ten times, and the results (Table 1) were presented as the IAP average value IAP_5FCV_ and its minimum and maximum values (min-max), which are estimates of the IAP confidence interval between 10% and 90%, are given. During the LOOCV procedure, the values of the number of positive examples (NP), the number of negative examples (NN), the number of correctly recognized positive examples (TP), the number of correctly recognized negative examples (TN), sensitivity = TP/NP, and specificity = TN/NN were also calculated. The result of the LOOCV procedure was used as a SAR model only if NP > 2 and the IAP exceeded the 95% confidence level. Finally, the 69 strain-specific models and one unspecified mutagenicity model were constructed. For 69 strain-specific models, the average IAP was about 0.944, and for unspecified mutagenicity it was 0.962 (see Table 1).

For a new compound, PASS calculates two probabilities for each activity: Pa (probability to be active (mutagenic)) and Pi (probability to be inactive (non-mutagenic)). A compound is considered predicted active if Pa > Pi. The PASS output probabilities (Pa, Pi) can be used to arrange the compounds. The IAP estimates the probability that a randomly chosen from the test dataset mutagen (active) will have a higher Pa than a randomly chosen non-mutagen (inactive). An important characteristic of the PASS algorithm is its robustness to training set imbalance. This algorithm has been specifically designed to handle highly imbalanced datasets, which can be essential when screening large chemical libraries where true actives represent only a small fraction among vast numbers of inactive compounds [8]. The algorithms of the PASS software differ significantly from many conventional SAR methods, which typically require balanced training sets. This requirement is difficult to satisfy when developing models for diverse activity types, such as strain-specific mutagenicity.

## 5. Conclusions

In summary, we have developed a comprehensive SAR modeling framework for Ames mutagenicity that spans 69 bacterial strains. The PASS algorithm has been implemented across more than 40 different computational resources available through the Way2Drug platform for predicting diverse properties of drug-like compounds from their structural formulas, ranging from biological activity spectra to potential metabolites and toxicological endpoints, including antiviral activity. The creation of this freely accessible web service aligns with open science principles and democratizes access to computational toxicology tools for researchers globally. Using PASS, we achieved robust predictive accuracy for most strains (average IAP 0.944). SAR models for individual strains varied in IAP value (from 0.8 to 1.0), largely in accordance with dataset size and chemical diversity. The high accuracy for many models indicates clear structure–activity relationship for mutagenic compounds across the dataset. These results emphasize the feasibility of reliable in silico Ames test predictions, potentially reducing experimental costs. Importantly, the large coverage of strains means that strain-specific mutagenic effects of compounds can be anticipated. The created SAR models were implemented as a new freely accessible application (Way2Drug Ames Mutagenicity predictor, https://way2drug.com/ames-mutagenicity/, (accessed on 1 December 2025) for the highly accurate prediction of compound mutagenicity across a comprehensive panel of 69 bacterial strains, enabling researchers and regulators to evaluate new chemicals by their structural formulae. Overall, this work demonstrates that advanced SAR modeling can complement experimental genotoxicity testing and support regulatory decision-making. The application is based on robust SAR models built with PASS software, demonstrating an average IAP of 0.944. Unlike conventional binary classifiers, this tool provides a detailed mutagenicity profile, offering insights into potential mechanisms of action and helping to identify strain-specific mutagenicity. This makes it an important resource for the early-stage safety assessment of chemicals in pharmaceutical, environmental, and regulatory contexts. By providing rapid, free, and detailed in silico mutagenicity screening to be integrated into modern chemoinformatics workflows, the Ames Mutagenicity Predictor aids in the prioritization of compounds for experimental testing, supports rational drug design, and accelerates the development of safer pharmaceutical molecules.

## Figures and Tables

**Table 1:** The characteristics of the SAR models for predicting Ames test mutagenicity for 69 strains.

Activity Type	NP	TP	NN	TN	Sen.	Sp.	IAP	IAP_5FCV_ (Min–Max)
Mutagenic (unspecified)	3250	2946	4285	3886	0.907	0.907	0.962	0.960 (0.960–0.961)
DJ400	4	4	7531	7169	1.000	0.952	0.983	0.979 (0.957–0.985)
DJ460	5	5	7530	7170	1.000	0.952	0.986	0.982 (0.967–0.988)
GW257	7	7	7528	7522	1.000	0.999	1.000	1.000 (1.000–1.000)
HISD3052	10	9	7525	7477	0.900	0.994	0.952	0.948 (0.916–0.964)
HISG46	19	16	7516	6757	0.842	0.899	0.925	0.905 (0.863–0.932)
SV50	4	4	7531	7470	1.000	0.992	0.995	0.990 (0.961–0.996)
TA100	2181	1856	5354	4552	0.851	0.850	0.912	0.910 (0.908–0.911)
TA100(NR)	68	63	7467	6788	0.927	0.909	0.960	0.956 (0.945–0.963)
TA100(PKM101)	3	3	7532	7528	1.000	1.000	1.000	1.000 (1.000–1.000)
TA100/1,8-DNP	20	17	7515	7013	0.850	0.933	0.946	0.930 (0.899–0.950)
TA100/GSH-	7	7	7528	7434	1.000	0.988	0.996	0.995 (0.993–0.996)
TA100/NG-57	3	3	7532	7492	1.000	0.995	0.998	0.988 (0.941–0.998)
TA100/TN5-1,8-DNP1011	4	3	7531	7519	0.750	0.998	0.917	0.900 (0.868–0.923)
TA100/TN5-1,8-DNP1012	5	4	7530	7516	0.800	0.998	0.933	0.921 (0.787–0.937)
TA102	206	162	7329	5868	0.786	0.801	0.875	0.867 (0.854–0.876)
TA103	3	3	7532	7497	1.000	0.995	0.997	0.992 (0.949–0.998)
TA104	137	110	7398	5930	0.803	0.802	0.876	0.871 (0.855–0.880)
TA1530	23	21	7512	6606	0.913	0.879	0.909	0.904 (0.890–0.914)
TA1535	467	386	7068	5865	0.827	0.830	0.902	0.897 (0.892–0.901)
TA1536	14	14	7521	7521	1.000	1.000	1.000	1.000 (1.000–1.000)
TA1537	394	335	7141	6076	0.850	0.851	0.913	0.909 (0.901–0.913)
TA1537(PKM101)	7	7	7528	7511	1.000	0.998	0.999	0.996 (0.967–1.000)
TA1538	311	261	7224	6040	0.839	0.836	0.911	0.908 (0.905–0.911)
TA1538(NR)	3	3	7532	7435	1.000	0.987	0.995	0.994 (0.990–0.995)
TA1950	4	3	7531	7468	0.750	0.992	0.921	0.922 (0.907–0.925)
TA1975	13	12	7522	6735	0.923	0.895	0.966	0.955 (0.920–0.972)
TA1977	26	26	7509	7270	1.000	0.968	0.997	0.995 (0.982–0.997)
TA1978	16	13	7519	6660	0.813	0.886	0.921	0.893 (0.861–0.937)
TA1978(PKM101)	5	4	7530	7501	0.800	0.996	0.936	0.832 (0.637–0.946)
TA2410	10	9	7525	6535	0.900	0.868	0.948	0.952 (0.950–0.956)
TA2637	46	45	7489	7252	0.978	0.968	0.991	0.991 (0.990–0.992)
TA2638	32	27	7503	6146	0.844	0.819	0.856	0.850 (0.834–0.863)
TA2638A	6	5	7529	5879	0.833	0.781	0.801	0.805 (0.791–0.814)
TA2662	3	3	7532	7497	1.000	0.995	0.997	0.992 (0.949–0.998)
TA4001	8	8	7527	7419	1.000	0.986	0.996	0.975 (0.815–0.996)
TA4006	11	10	7524	6874	0.909	0.914	0.946	0.945 (0.924–0.954)
TA7001	14	13	7521	7328	0.929	0.974	0.980	0.979 (0.974–0.981)
TA7002	25	20	7510	7185	0.800	0.957	0.925	0.923 (0.918–0.927)
TA7003	11	11	7524	7370	1.000	0.980	0.993	0.992 (0.985–0.993)
TA7004	30	23	7505	5396	0.767	0.719	0.851	0.850 (0.824–0.859)
TA7005	29	23	7506	6587	0.793	0.878	0.911	0.908 (0.902–0.918)
TA7006	21	17	7514	7234	0.810	0.963	0.908	0.905 (0.896–0.910)
TA92	23	18	7512	6682	0.783	0.890	0.881	0.876 (0.819–0.891)
TA97	239	186	7296	5667	0.778	0.777	0.857	0.850 (0.838–0.859)
TA97A	64	50	7471	5813	0.781	0.778	0.862	0.855 (0.838–0.865)
TA98	1727	1495	5808	5026	0.866	0.865	0.929	0.927 (0.926–0.929)
TA98(NR)	171	156	7364	6707	0.912	0.911	0.965	0.964 (0.961–0.965)
TA98/1,8-DNP6	164	151	7371	6706	0.921	0.910	0.967	0.966 (0.965–0.968)
TM677	13	12	7522	6578	0.923	0.875	0.945	0.944 (0.926–0.955)
TM677/8-azaguanine	6	6	7529	7090	1.000	0.942	0.987	0.911 (0.696–0.990)
YG1012	9	8	7526	6423	0.889	0.853	0.934	0.925 (0.908–0.941)
YG1019	16	15	7519	6996	0.938	0.930	0.966	0.966 (0.959–0.972)
YG1020	23	22	7512	6946	0.957	0.925	0.978	0.975 (0.965–0.980)
YG1021	42	38	7493	6997	0.905	0.934	0.974	0.973 (0.970–0.975)
YG1024	84	77	7451	6849	0.917	0.919	0.968	0.962 (0.954–0.970)
YG1025	18	18	7517	6931	1.000	0.922	0.983	0.981 (0.969–0.985)
YG1026	38	36	7497	6964	0.947	0.929	0.986	0.984 (0.980–0.987)
YG1029	57	51	7478	6887	0.895	0.921	0.963	0.955 (0.943–0.964)
YG1041	7	6	7528	6992	0.857	0.929	0.888	0.891 (0.871–0.906)
YG1042	8	8	7527	6620	1.000	0.880	0.972	0.955 (0.892–0.975)
YG7104	7	5	7528	7462	0.714	0.991	0.908	0.906 (0.873–0.910)
YG7104ER	3	3	7532	7530	1.000	1.000	1.000	1.000 (1.000–1.000)
YG7108	8	7	7527	6047	0.875	0.803	0.892	0.885 (0.833–0.897)
YG7108-2A6	3	3	7532	7418	1.000	0.985	0.989	0.990 (0.988–0.991)
YG7108-2A6/OR	3	3	7532	7418	1.000	0.985	0.989	0.990 (0.988–0.991)
YG7108ER	3	3	7532	7530	1.000	1.000	1.000	1.000 (1.000–1.000)
YG7112	10	6	7525	6204	0.600	0.825	0.823	0.816 (0.769–0.835)
YG7113	10	6	7525	6204	0.600	0.825	0.823	0.816 (0.769–0.835)
BD190 REC *	3	3	7532	7530	1.000	1.000	1.000	1.000 (1.000–1.000)
Mean value					0.896	0.920	0.944	0.937 (0.933–0.943)

NP is the number of positive examples. TP is the number of correctly recognized positive examples using the LOOCV procedure. NN is the number of negative examples. TN is the number of correctly recognized negative examples using the LOOCV procedure. Sen. is the sensitivity, TP/NP. Sp. is the specificity, TN/NN. IAP is value of the Invariant Accuracy of Prediction using the LOOCV procedure. IAP_5FCV_ is the average value of the Invariant Accuracy of Prediction and its minimum and maximum (min–max) values for ten repetitions of the 5-fold CV procedure. *—BD190 REC is *Bacillus subtilis* strain, other belong to *Salmonella typhimurium* strains.

## Data Availability

The data presented in this study are available on request from the corresponding author. A web interface implementing the model is available at https://way2drug.com/ames-mutagenicity/ (accessed on 1 December 2025) for prediction using chemical structures, entering a SMILES string, or conducting searches based on a common compound name.

## Abbreviations

The following abbreviations are used in this manuscript:

5FCV	5-fold cross-validation
IAP	Invariant Accuracy of Prediction
ICH M7	International Council for Harmonization guideline M7
LOOCV	Leave-one-out cross-validation
MNA	Multilevel Neighborhoods of Atoms
OECD	Organization for Economic Co-operation and Development
PASS	Prediction of Activity Spectra for Substances
Pa	probability to be active
Pi	probability to be inactive
QSAR	Quantitative Structure-Activity Relationship
SAR	Structure-Activity Relationship
SMILES	Simplified Molecular Input Line Entry System
